# A forced cough sound based pulmonary function assessment method by using machine learning

**DOI:** 10.3389/fpubh.2022.1015876

**Published:** 2022-10-25

**Authors:** Wenlong Xu, Guoqiang He, Chen Pan, Dan Shen, Ning Zhang, Peirong Jiang, Feng Liu, Jingjing Chen

**Affiliations:** ^1^College of Information Engineering, China Jiliang University, Hangzhou, Zhejiang, China; ^2^The First Affiliated Hospital, College of Medicine, Zhejiang University, Hangzhou, Zhejiang, China; ^3^Lishui People's Hospital, Lishui, Zhejiang, China; ^4^School of Information Technology and Electrical Engineering, University of Queensland, Brisbane, QL, Australia; ^5^Department of Digital Urban Governance and School of Computer and Computing Science, Zhejiang University City College, Hangzhou, China

**Keywords:** forced cough sound, pulmonary function assessment, machine learning, digital medical, mobile service

## Abstract

Pulmonary function testing (PFT) has important clinical value for the early detection of lung diseases, assessment of the disease severity, causes identification of dyspnea, and monitoring of critical patients. However, traditional PFT can only be carried out in a hospital environment, and it is challenging to meet the needs for daily and frequent evaluation of chronic respiratory diseases. In this study, we propose a novel method for accurately assessing pulmonary function by analyzing recorded forced cough sounds by mobile device without time and location restrictions. In the experiment, 309 clips of cough sound segments were separated from 133 patients who underwent PFT by using Audacity software. There are 247 clips of training samples and 62 clips of testing samples. Totally 52 features were extracted from the dataset, and principal component analysis (PCA) was used for feature reduction. Combined with biological attributes, the normalized features were regressed by using machine learning models with pulmonary function parameters (i.e., FEV1, FVC, FEV1/FVC, FEV1%, and FVC%). And a 5-fold cross-validation was applied to evaluate the performance of the regression models. As described in the experimental result, the result of coefficient of determination (R2) indicates that the support vector regression (SVR) model performed best in assessing FVC (0.84), FEV1% (0.61), and FVC% (0.62) among these models. The gradient boosting regression (GBR) model performs best in evaluating FEV1 (0.86) and FEV1/FVC (0.54). The result confirmed that the proposed method was capable of accurately assessing pulmonary function with forced cough sound. Besides, the cough sound sampling by a smartphone made it possible to conduct sampling and assess pulmonary function frequently in the home environment.

## Introduction

According to the World Health Organization's survey on common respiratory diseases, by 2021, Chronic Obstructive Pulmonary Disease (COPD) and related diseases caused by air pollution had caused about 7 million deaths every year. It is estimated that by 2030, global COPD will become the third leading cause of death (1). In particular, with a large population, China is undergoing a considerable burden of respiratory diseases. Wang et al. reported that in 2018, there were nearly 100 million patients with COPD in China (2), which shows that respiratory diseases have been widely spread in China. Pulmonary function testing (PFT) is the gold standard for the clinical evaluation of respiratory diseases (3). In clinical practice, PFT can feedback on the abnormal lung status of patients according to their pulmonary function parameters, which is the primary approach assisting doctors in diagnosing respiratory diseases (4). The patient is required to put his/her mouth on the bite of the spirometer, inhaled as deeply as possible, and then exhaled hard to expel all the air as quickly as possible to fulfill the pulmonary ventilation function testing. The patient must fully complete this process, exert maximum inspiratory and expiratory force, and repeat this process until three consistent measurements are obtained (5). According to the patient's age, gender, height, and weight, the reference values of 1-s forced expiratory flow (FEV1), forced vital capacity (FVC), and 1-s expiratory rate (FEV1/FVC) of each patient are estimated. The ratios between the final measured pulmonary function value and the reference of the patient's, FEV1%, and FVC% are the indicators of the severity of respiratory disease (6, 7) (Table 1). FEV1/FVC and FVC% help distinguish obstructive, restrictive, and normal respiratory patterns. The severity of obstructive diseases can be determined by FEV1% (8).

**Table 1:** Description of pulmonary function parameters.

**Term**	**Description**
FEV1	Forced expiratory volume in one second
FVC	Forced vital capacity
FEV1/FVC	Forced expiratory volume in one second / Total vital capacity
FEV1%	Measured FEV1 value / Reference FEV1 value
FVC%	Measured FVC value / Reference FVC value

High-cost and complex operation procedures prevent the wide adoption of the traditional PFT method. Pulmonary function departments are available only in large hospitals. The PFT is still unreachable in large-scale respiratory disease screening but is a frequent requirement for chronic respiratory disease patients (9). The situation is even worse in low-income areas where chronic respiratory disease is usually more prevalent (10). Therefore, new methods, which are easy to use and low-cost, are urgently expected. A typical process of cough can be divided into three stages: (1) inhalation, (2) compression, and (3) exhalation (11). It contains some similar procedures to pulmonary ventilation function testing. A smartphone can record cough sounds and transmit the data to the Internet easily. Such a procedure for data collection is non-invasive, touchless, and can be completed at home without any professional staff. The duration of the cough (12) is shorter than that of pulmonary ventilation function testing, which usually lasts about 6 s (13). Cough is an important early symptom of respiratory diseases (14). As a protective physiological reflex action, cough is affected by acoustic characteristics of airflow, tissue, and the shape of the lung and airway (15). Due to its unique histology and organ pathology, different respiratory diseases show characteristic features in cough. The cough sounds of patients with pneumonia and asthma were proved to be different (16). However, cough sounds have not been widely adopted to estimate pulmonary function. It is promising in discriminating respiratory diseases in clinical, disease prevention, and control. In this study, we proposed a novel method for assessing pulmonary function parameters based on cough sounds collected by mobile devices. The major contributions in this study are summarized as follows.

- We introduced the cough sound to construct a touchless, non-invasive method to assess pulmonary function parameters that meet the requirement of daily monitoring for patients with chronic respiratory diseases.
- We adopted multiple regression models to predict five pulmonary function parameters, refine the learning process of pulmonary function parameters, and improve the prediction accuracy.
- We used 309 clips of cough sounds collected from 133 subjects in the same environment and with homogeneous criteria. The data of patients with different severity of lung disease were adopted to improve the generalization ability of the proposed model.

The structure of this article is organized as follows. In section related work, we present an overview of related works. In section materials and methods, we introduce the detailed process of the proposed method. In section results, we introduce the experimental results. In section conclusion, we conclude this study and discuss future research.

## Related work

### Assessment of pulmonary function based on sound signals

Sound has been widely used for pulmonary function assessment recently. Compared with the traditional PFT method, the diagnosis and evaluation of respiratory diseases based on sound signals is not only convenient but also low cost. Alam et al. (17) developed three prediction models based on speech and breathing sound signals. Through 323 clips of speech and breathing sounds of 26 subjects, five features (spectral contrast, rolling at 95%, root mean square energy, spectral bandwidth, and average amplitude) were extracted, and the random forest regression model was used to train and predict the pulmonary function parameter FEV1%, and it achieved an RMSE of 10.86 and an MAE of 11.47. A support vector machine model was used to classify the severity of four kinds of pulmonary function, and the accuracy was 73.20%, and an accuracy of 85% was achieved to judge whether the subjects had abnormal pulmonary function through the random forest classification model. In addition, Nazir (18) adopted mobile devices for the diagnosis of chronic respiratory diseases, and 201 subjects were enrolled to collect “A-vowel” sound or “AAAA...” sound to assess the pulmonary function parameter FEV1/FVC by using the multi-layer regression model, and it achieved an MAE of 7.4%. Moreover, keuml (19) used mobile phones to collect the speech sound signals of 59 subjects and proposed two algorithms for passive evaluation of pulmonary function: the first one used a random forest classifier model to distinguish whether the subjects were healthy or had obstructive respiratory disease, and obtained an accuracy of 78.6%; the latter one used the 7-dimension features of speech sounds by neural network model to assess FEV1/FVC pulmonary function parameters, and achieved an MAE of 12.5%.

### Assessment of pulmonary function based on cough sounds

Cough is a common symptom in a variety of respiratory diseases (20). Respiratory diseases that affect the human body will promote secretion in the airway. As a protective response, this secretion will cause patients to cough. Clinical investigation indicates that the severity of cough is an important indicator to understand the progress of respiratory diseases (21). The features of cough sound include the description of the respiratory system. The features extracted by signal processing technology can be used to establish an effective disease assessment and diagnostic method. However, compared with other respiratory disease diagnosis methods based on sound signals (such as wheezing, speech, and vowels), the use of cough sound for estimation has not attracted wide attention yet. Windmon (22) focuses on using cough sound signals to evaluate and diagnose COPD. By using 13 spectral features extracted from cough sounds of 23 COPD patients and 16 healthy subjects, the random forest classification model was used to train these samples and an accuracy of 85.6% was achieved. Hee (23) tried to establish a classification model using cough sounds to analyze whether children have asthma. Mel Frequency Cepstrum Coefficient (MFCC) and Constant-Q Cepstral Coefficients (CQCC) signal processing techniques were used to extract the features of 1,192 clips of cough sounds from 89 children with asthma and 1,140 clips of cough sounds from healthy children. Gaussian mixture model (GMM) classification was used to train the samples, and the specificity was 82.81% and the sensitivity was 84.76%.

With the wide adoption of cough sound in the diagnosis of respiratory diseases, a lot of literature focus on the study of cough sound on pulmonary function parameters. According to Achuth (24) study, the cough sound signal can better predict pulmonary function parameters than the wheeze sound signal. Cough and wheeze were recorded in 16 healthy people and 12 patients, and statistical spectrum description (SSD) was used as the cue. Support vector regression (SVR) was used to predict FEV1%, FVC%, and FEV1/FVC pulmonary function parameters, and achieve RMSE of 11.06, 10.3, and 0.08. Moreover, the severity of asthma was also classified and evaluated, with an accuracy of 77.77%. More subjects were achieved in Sharan's (25) study, cough sounds from 322 adults were collected to estimate FEV1, FVC, and FEV1/FVC with support vector expression and a random forest model, reaching RMSE of 0.593, 0.725, and 0.164.

### Application of cough sound in the epidemic period of COVID-19

COVID-19 can be detected by cough sound (26). As reported by MIT (27), an artificial intelligence speech processing framework was developed, and COVID-19 was screened from cough sounds by using the processing feature extractor of cough sound signal. The convolutional neural network model was trained by 4,256 subjects' cough sounds and tested by 1,064 subjects, and the sensitivity and specificity of COVID-19 detected by this model were 98.5 and 94.2%. For the asymptomatic subjects, the model achieved a sensitivity of 100% and a specificity of 83.2%.

In addition, cough sounds can also assist in the online screening of respiratory diseases during the epidemic of COVID-19. Due to the normalization of COVID-19, doctors' clinical diagnosis is limited to reducing the spread of the virus. Therefore, online system assisted with remote diagnosis has attracted more and more attention. The symptom detection model for early respiratory diseases will be a solution that can be implemented on low-power mobile devices to replace the preliminary screening of health practitioners to reduce the risk of infection transmission. For example, online medical consultation methods have emerged based on cough sounds in recent years. In Nemati's (28) study, the application in the mobile phone was used to collect cough sounds, and the patient's current status of pulmonary obstruction disease was feedback through the Internet. The estimated MAE of pulmonary obstruction as COPD and asthma were 8 and 9%, respectively. Kosasih et al. (29) proposed a cough detection method for multiple respiratory diseases, which analyzes the sound of cough in an AI model. This method used multiple classifiers (such as LR, ANN, SVM, and RF), and achieved a sensitivity of 86%, specificity of 91%, and accuracy of 91%. Sharma (30) and Chowdhury (31) realized the detection of subjects' infection with COVID-19 by recording cough sounds through mobile phones, and the classification accuracy was 66.74% and the sensitivity was 92.77%.

## Materials and methods

**Figure 1:**
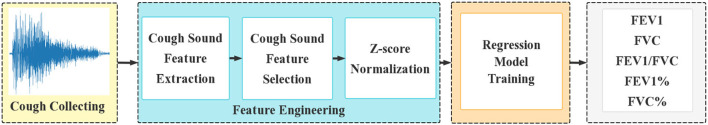
The procedures for assessing pulmonary function parameters.

The regression model for PFT is detailed in Figure 1. Cough sound segmentation was handled with Audacity (32) on cough sound clips sampled by a smartphone. For each single cough sound, multi-dimensional features were extracted. The features were optimized using principal component analysis (PCA) (33) and then normalized through Z-score normalization (34). The trained result of the five pulmonary function parameters was used as input in the regression model.

### Dataset collection

This study (including the protocols and subject recruitments) was approved by the human ethics committees of Lishui people's Hospital in China. In the experiment, a total of 133 subjects were recruited to complete the PFT, in which, the cough sounds were recorded within 10 min. The distribution of subjects and their demographic information are shown in Table 2. A mobile application developed by China Jiliang University, paired with a smartphone, HONOR 60, was used to collect the subject's cough sounds. The smartphone was placed ~40 cm away from the mouth of the subject at an angle of roughly 45°. The sounds were recorded under the onsite instruction. And the sampling frequency was 16,000 Hz. Each subject was instructed to cough at least three times within 30 s, and the interval between the consecutive coughs was 1 s. It is emphasized that during the experiment, except for the cough sound, age, height, weight, and gender of the subjects, this study did not collect any personal information of the subjects.

**Table 2:** Statistical overview of demographic and cough data.

	**Normal**	**Mildly**	**Moderately**	**Severely**	**Total**
Number of subjects	69	29	27	8	133
Number of coughs	170	72	55	12	309
Age in years (SD)	64.41 (16.04)	53.29 (15.81)	70.07 (11.01)	80.38 (6.87)	60.75 (17.51)
Gender (Male/ Female)	43:26	16:13	20:7	7:1	86:47
Height (cm)	162.99	161.03	162.11	163.00	162.38
Weight (kg)	63.65	62.31	70.48	57.13	64.35

The experimental result showed that 69 subjects tested normal, 29 subjects tested mildly abnormal (including 9 obstructive cases, 5 restricted cases, and 15 mixed cases), 27 subjects tested moderately abnormal (including 7 obstructive cases and 20 mixed cases), the other 8 subjects tested severely abnormal (including one obstructive case and 7 mixed cases). The above assessment results were obtained based on the latest guidelines for PFTs (35) in 2021. Using Audacity, 309 clips of single cough sounds with a time duration of 350 ms were extracted from 133 cough sound files. Each cough sound was annotated with pulmonary function parameters (i.e., FEV1, FVC, FEV1/FVC, FEV1%, FVC%) and biological attributes (i.e., age, sex, height, and weight). The ratio of the training set to the testing set was 8:2. The 309 clips of cough sounds were randomly divided into the training set (247) and the testing set (62).

### Feature engineering

#### Feature extraction

In this study, we used a mixture of traditional features and novel features (36, 37), and features were generated by using the Librosa toolkit in Python, which was widely used for acoustic analysis (38). The 52-dimensions features in the time domain and frequency domain were extracted to map the relationship between cough features and pulmonary function parameters. The main features include Mel-frequency cepstral coefficient (MFCC), zero-crossing rate (ZCR), signal energy, spectral features (spectral centroid, spectral bandwidth, spectral roll-off), and calculated hue centroid features. These 52 features are time and frequency features extracted by the solid sound signal processing method and this feature was taken due to its comprehensiveness and paralinguistics (39). The 52 features used in this article are from Gowrisree's study, which objectively describes the primary and secondary characteristics of cough sound. It also describes the impact of primary and secondary features of cough sounds on the clinical diagnosis of lung function. Besides, the 52 features extracted in this article provide a complete description of the information in the time domain and frequency domain of the cough sound signal. We attempted to use these features for fitting the parameters of lung function. In addition, four biological attributes of the subjects were also used. This is because the reference value of pulmonary function parameters was evaluated and calculated through biological attributes, which could describe the relationship between cough sound and pulmonary function parameters through the biological attributes of subjects. Table 3 shows the specific descriptions of the 52 cough-sound features and 4 biological attributes.

**Table 3:** Statistical overview of features.

**Features**	**Number**	**Explained in detail**
MFCC	1–40	A Mel scale is used to tone the obtained pitch and frequency to the actual measured frequency.
Zero-crossing rate	41	Determine the number of times a signal crosses the zero amplitude line.
RMS	42	The square root of the signal is used to characterize the energy in the signal.
Spectral contrast	43–49	The decibel difference between peaks and valleys in the spectrum.
Spectral centroid	50	Indicates the center of mass of the spectrum is located.
Spectral bandwidth	51	The bandwidth of light is at one-half the peak maximum.
Spectral roll-off	52	The frequency below which a specified percentage of the total spectral energy.
Patient details	53–56	Age, Gender, Height, Weight.

#### Feature selection

Among 52 cough sound features and 4 biological features, none has an equivalent value in evaluating pulmonary function parameters. By PCA, observations of correlated variables were converted to a set of linearly uncorrelated orthogonal variables, which were ordered in the way that each orthogonal variable has the largest possible variance under the constraint of being orthogonal to all preceding components (40). In this way, the number of features was reduced while preserving as much information as possible. More precisely, PCA mapped the high-dimensional space $X={\left[x_{1},x_{2},\cdots \;,x_{i}\right]}^{T}$ to a low-dimensional space Y, and searched a maximum value of linear mapping by **Formula (1)** (41).


(1) $$\begin{array}{l} Y\;=\mathrm{argmax}\;Tr\left(W^{T}Cov\left(X\right)W\right) \end{array}$$

In **Formula (1)**, *Cov*(*X*) represents a covariance matrix of the data X. W represents a transformation matrix of X and WT represents the transposed matrix of W. Tr(X) denotes the trace of an n-by-n square matrix of the X. The *argmax* represents the maximum value of linear mapping.

#### Feature normalization

With regard to different scales, features were normalized to eliminate the influence of scale differences. **Formula (2)** was used to scale the values in each feature to a mean of 0 and a variance of 1.


(2) $$\begin{array}{l} X\left(i\right)=\frac{X\left(i\right)\;-\;\bar{X}}{\sigma } \end{array}$$

In **Formula (2)**, σ represents the standard deviation of a feature and $\bar{X}$ denotes the mean value of a feature.

### Regression model and evaluation indicators

In this study, seven regression models were taken for performance comparison, including support vector regression (SVR), random forest regression (RF), Bayesian ridge regression (BRD), gradient enhanced regression (GBR), ridge regression (RD), extreme learning machine (ELM), and multi-layer perceptron (MLP). The parameters of each model were automatically adjusted using the gray wolf optimization (GWO) algorithm (42). Five pulmonary function parameters (FEV1, FVC, FEV1/FVC, FEV1%, and FVC%) were assessed. Each of these parameters was compared with seven regression models. In the training process, 5-fold cross-validation was used to evaluate the performance of the model. Three indicators were used to evaluate the accuracy of the regression model, including root mean square error (RMSE), mean absolute error (MAE), and coefficient of determination (R2).


(3) $$\begin{array}{l} RMSE=\sqrt{\frac{1}{m}\sum_{i=1}^{m}{\left(yi-\ddot{yi}\right)}^{2}} \end{array}$$

(4) $$\begin{array}{l} MAE=\frac{1}{m}\sum_{i=1}^{m}\left|yi-\ddot{yi}\right| \end{array}$$

(5) $$\begin{array}{l} R^{2}=1-\frac{\sum_{i}{\left(\ddot{yi}-yi\right)}^{2}\;}{\sum_{i}{\left(\bar{yi}-yi\right)}^{2}} \end{array}$$

In **Formulas (3), (4), and (5)**, $\bar{yi}$ represents the estimated value, $\ddot{yi}$ represents the real value, $\bar{yi}$ represents the mean of real value, and *m* represents the number of samples.

## Results

The accuracy of the proposed model with biological attributes was compared with the result of the same model without biological attributes. The root mean square error (RMSE), mean absolute error (MAE), and coefficient of determination (*R*^2^) were taken as evaluation indexes.

### Experimental results

As shown in Table 4, the accuracy of seven regression models in evaluating five pulmonary function parameters with regard to features of the cough sounds and biological attributes was listed. Each parameter was analyzed by seven regression models, and the best-performance model was selected to complete the evaluation. As illustrated in Table 4, the evaluation of the best-performance model depended on the effect of the coefficient of determination (*R*^2^).

**Table 4:** Performance comparison of seven regression models.

**Model**	**Indicators**	**FEV1**	**FVC**	**FEV1/FVC**	**FEV1%**	**FVC%**
SVR	RMSE	0.41	0.38	0.11	15.63	11.27
	MAE	0.33	0.31	0.09	11.55	8.71
	R^2^	0.82	0.84	0.45	0.61	0.62
RF	RMSE	0.35	0.42	0.13	15,10	13.17
	MAE	0.28	0.30	0.10	11.67	10.46
	R^2^	0.81	0.81	0.38	0.44	0.42
GBR	RMSE	0.38	0.44	0.11	20.42	14.56
	MAE	0.27	0.34	0.08	15.49	10.88
	R^2^	0.86	0.81	0.54	0.34	0.37
RD	RMSE	0.44	0.49	0.12	18.80	13.49
	MAE	0.36	0.41	0.10	15.05	10.61
	R^2^	0.74	0.76	0.26	0.28	0.16
BRD	RMSE	0.48	0.54	0.11	17.27	16.77
	MAE	0.40	0.43	0.09	13.52	13.50
	R^2^	0.75	0.76	0.29	0.35	0.17
MLP	RMSE	0.47	0.48	0.22	25.53	20.61
	MAE	0.38	0.39	0.17	20.86	15.87
	R^2^	0.70	0.77	– 1.54	– 0.18	0.25
ELM	RMSE	0.54	0.64	0.15	18.70	16.97
	MAE	0.42	0.51	0.12	14.52	13.82
	R^2^	0.68	0.58	0.13	0.45	0.15

The values with bold and underline are the best values.

For the evaluation of **FEV1**, gradient enhanced regression (GBR) is the model with the best performance, and its accuracy is RMSE: 0.38, MAE: 0.27, and *R*^2^: 0.85.

For the evaluation of **FVC**, support vector regression (SVR) is the model with the best performance, and its accuracy is RMSE: 0.38, MAE: 0.31, and *R*^2^: 0.84.

For the evaluation of **FEV1/FVC**, gradient enhanced regression (GBR) is the model with the best performance, and its accuracy is RMSE: 0.11, MAE: 0.08, and *R*^2^: 0.53.

For the evaluation of FEV1%, support vector regression (SVR) is the model with the best performance, and its model accuracy is RMSE: 15.63, MAE: 11.55, and *R*^2^: 0.61.

For the evaluation of FVC%, support vector regression (SVR) is the model with the best performance, and its accuracy is RMSE: 11.27, MAE: 8.71, and *R*^2^: 0.62.

**Figure 2:**
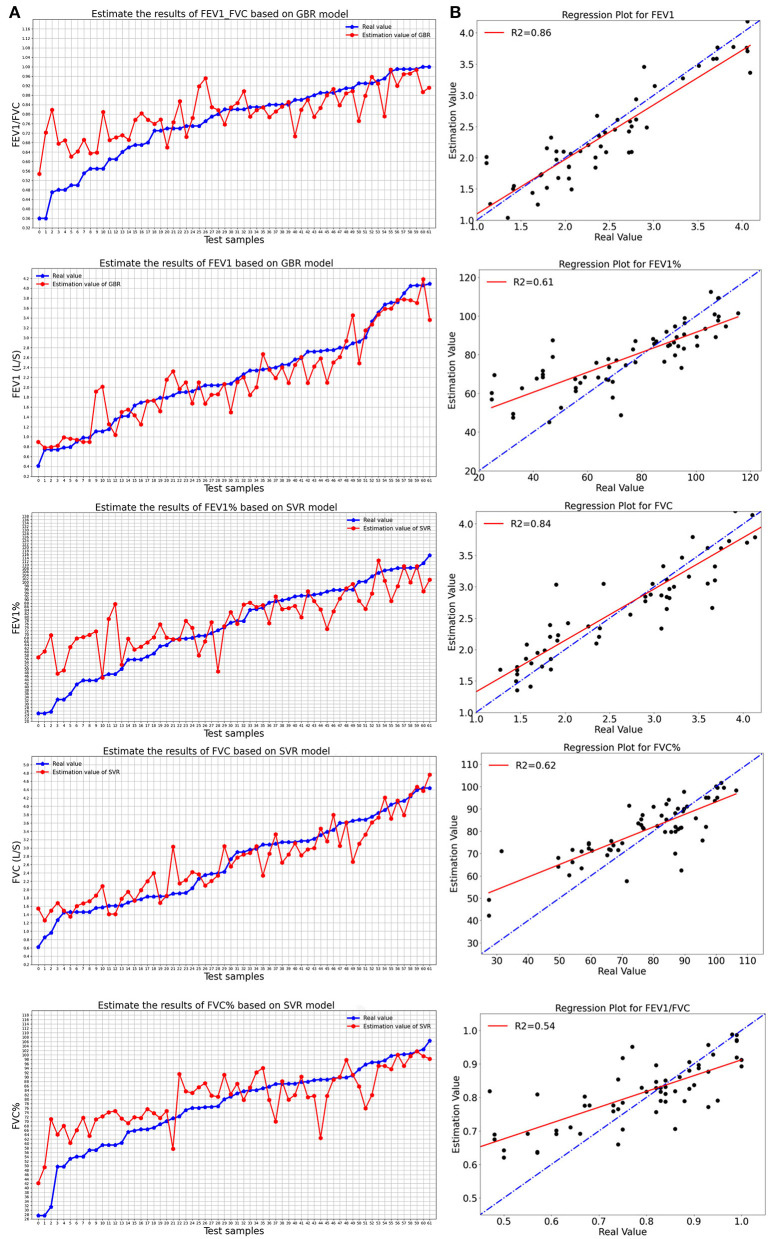
The best-performance model evaluated the results of five pulmonary function parameters: **(A)** Comparison between real value and estimated value of the best model; **(B)** Regression diagram of pulmonary function parameters of the best-performance model.

**Figure 3:**
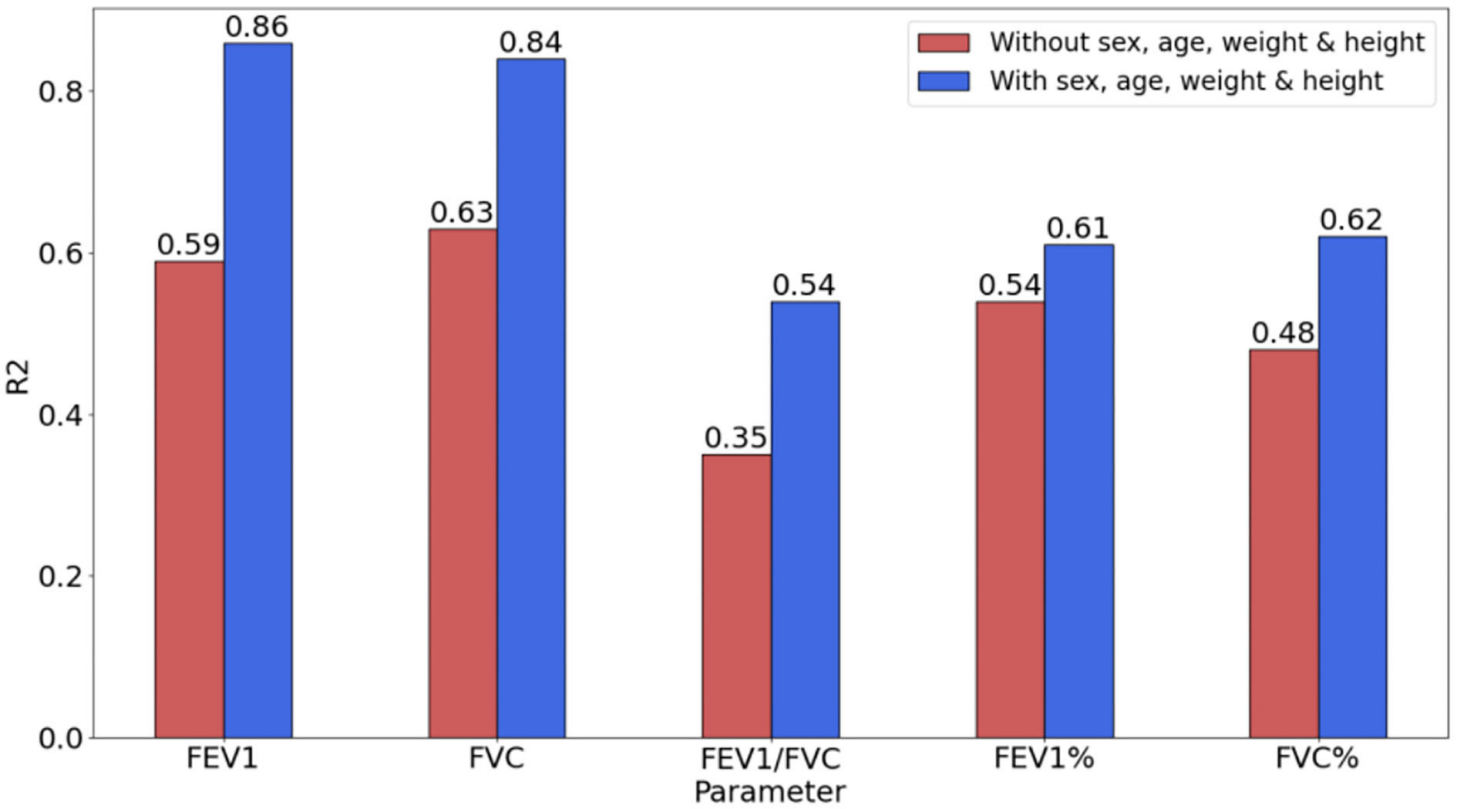
Performance comparison of the model with/without biological attributes through R2.

Figure 2 shows the evaluation results of the best-performance models of five pulmonary function parameters and their regression diagrams. Figure 2A compares the estimated value and the real value of pulmonary function parameters obtained using the optimal model. Figure 2B shows the regression diagram and the corresponding test results of the best model. Besides, the experimental results of the remaining estimation regression models are shown in Figure 3.

### Effects of biological features

To verify the impact of biological attributes while evaluating pulmonary function parameters, the evaluation indicators (with/without biological attributes) based on the SVR model were compared. The biological attributes include the subjects' age, sex, weight, and height, which is an important basis for constituting the reference value of the pulmonary function.

Table 5 shows the impact of biological attributes on the accuracy of the regression model. The introduction of biological attributes caused the decrease in RMSE and MAE. For the regression model with biological attributes, the improved RMSE for FEV1, FVC, FEV1, and FVC% were 0.23,0.20,1.39, and 1.98, respectively. The FEV1/FVC did not decrease, but the MAE was improved by 0.01.

**Table 5:** Performance comparison of the model with/without biological attributes based on SVR.

**Model**	**Indicators**	**FEV1**	**FVC**	**FEV1/FVC**	**FEV1%**	**FVC%**
Model (SVR) with sex,	RMSE	0.38	0.38	0.11	15.63	11.27
age, weight & height	MAE	0.27	0.31	0.08	11.55	8.71
	R^2^	0.86	0.84	0.54	0.61	0.62
Model (SVR) with sex,	RMSE	0.61	0.58	0.11	17.02	13.25
age, weight & height	MAE	0.49	0.44	0.09	13.12	10.48
	R^2^	0.59	0.63	0.35	0.54	0.48

The values with bold and underline are the best values.

As shown in Figure 3, *R*^2^ was significantly improved by introducing biological attributes. The improvement in *R*^2^ for FEV1, FVC, FEV1/FVC, FEV1, and FVC% were 0.27, 0.21, 0.19, 0.07, and 0.14, respectively.

## Conclusion

In this study, we introduced sound features and biological attributes to predict the pulmonary function parameters by using a group of machine learning models. The extracted features were normalized and dimension reduced by PCA, and GWO was adopted to adjust the parameters of the model automatically. An experiment including 133 subjects was conducted to validate the effectiveness of the proposed method for PFT. The result showed that the method can accurately predict pulmonary function. However, there are still some limitations, which should be further studied in future. In the proposed model, similar subject cough sounds may cause a correlation to impact the accuracy of the model. A better way to integrate all the results from multiple attempts by the same subject is expected. In addition, this study was conducted in a controlled environment. It will consider subject changes to improve the robustness of the model. In summary, the proposed method can be easily applied in smartphone, providing a convenient and non-invasive way to assess pulmonary function.

## Data availability statement

The raw data supporting the conclusions of this article will be made available by the authors, without undue reservation.

## Ethics statement

The studies involving human participants were reviewed and approved by Lishui People's Hospital. Written informed consent for participation was not required for this study in accordance with the national legislation and the institutional requirements.

## Author contributions

WX and GH proposed the research plan and manuscript draft. JC, WX, and GH completed the final manuscript for submission. All the authors worked together to complete the algorithm development and carry out the experiment. All authors contributed to the article and approved the submitted version.

## Funding

This work was supported by the Natural Science Foundation of China (No. 61672476), the Innovation and Entrepreneurship Training Project for National Undergraduates (No. 202110356070S), and Key Research and Development projects of Zhejiang Province (No. 2020ZJZC02).

## Conflict of interest

The authors declare that the research was conducted in the absence of any commercial or financial relationships that could be construed as a potential conflict of interest.

## Publisher's note

All claims expressed in this article are solely those of the authors and do not necessarily represent those of their affiliated organizations, or those of the publisher, the editors and the reviewers. Any product that may be evaluated in this article, or claim that may be made by its manufacturer, is not guaranteed or endorsed by the publisher.
